# Isolating the impact of antipsychotic medication on metabolic health: Secondary analysis of a randomized controlled trial of antipsychotic medication versus placebo in antipsychotic medication naïve first‐episode psychosis (the STAGES study)

**DOI:** 10.1111/eip.13353

**Published:** 2022-10-04

**Authors:** Brian O'Donoghue, Kelly Allott, Susy Harrigan, Franco Scalzo, Janine Ward, Sumudu Mallawaarachchi, Sarah Whitson, Lara Baldwin, Jessica Graham, Edward Mullen, Craig MacNeil, Dylan Alexander, Stephen J. Wood, Michael Berk, Mario Alvarez‐Jimenez, Andrew Thompson, Alex Fornito, Hok Pan Yuen, Barnaby Nelson, Shona M. Francey, Patrick McGorry

**Affiliations:** ^1^ Centre for Youth Mental Health University of Melbourne Victoria Australia; ^2^ Orygen Parkville Victoria Australia; ^3^ Department of Social Work, School of Primary and Allied Health Care Monash University Melbourne Australia; ^4^ Centre for Mental Health, School of Population and Global Health The University of Melbourne Parkville Victoria Australia; ^5^ Turning Point, Addiction Treatment Centre Richmond Victoria Australia; ^6^ Melbourne School of Psychological Sciences The University of Melbourne Parkville Victoria Australia; ^7^ School of Psychology University of Birmingham Edgbaston UK; ^8^ School of Medicine IMPACT Strategic Research Centre, Deakin University Geelong Australia; ^9^ Division of Mental Health and Wellbeing, Warwick Medical School University of Warwick Coventry UK; ^10^ Brain Mind and Society Research Hub, Turner Institute for Brain and Mental Health, School of Psychological Sciences Monash University Melbourne Australia

**Keywords:** antipsychotic medication, metabolic syndrome, obesity, psychosis, schizophrenia

## Abstract

**Background:**

Cardiovascular and metabolic diseases are the leading contributors to the early mortality associated with psychotic disorders. To date, it has not been possible to disentangle the effect of medication and non‐medication factors on the physical health of people with a first episode of psychosis (FEP). This study aimed to isolate the effects of antipsychotic medication on anthropometric measurements, fasting glucose and lipids.

**Methods:**

This study utilized data from a triple‐blind randomized placebo‐controlled trial comparing two groups of antipsychotic‐naïve young people with a FEP who were randomized to receive a second‐generation antipsychotic medication (FEP‐medication group) or placebo (FEP‐placebo group) for 6 months. Twenty‐seven control participants were also recruited.

**Results:**

Eighty‐one participants commenced the trial; 69.1% completed at least 3 months of the intervention and 33.3% completed the full 6 months. The FEP‐placebo group gained a mean of 2.4 kg (±4.9) compared to 1.1 kg (±4.9) in the control participants (*t* = 0.76, *p* = .45). After controlling for multiple analyses, there was no difference in blood pressure, waist circumference or heart rate between the FEP‐placebo group and controls. After 6 months, the FEP medication group had gained 4.1 kg (±4.5), higher than those receiving placebo but not statistically significant (*t* = 0.8, *p* = .44). There were no differences in fasting glucose or lipids between the FEP groups after 3 months.

**Conclusions:**

While limited by small numbers and high attrition, these findings indicate that some of the metabolic complications observed in psychotic disorders could be attributable to factors other than medication. This emphasizes the need to deliver physical health interventions early in the course of FEP.

## 1 BACKGROUND

Second‐generation antipsychotic medications are the first‐line treatment for psychotic disorders; however, they are associated with significant metabolic side‐effects (Leucht et al., 2013). These side‐effects result in higher rates of obesity, diabetes mellitus, and cardiovascular disease in this population (Annamalai et al., 2017), which contribute to a reduced life expectancy of approximately 15 years (Hjorthøj et al., 2017). However, individuals presenting with a first episode of psychosis (FEP) also have a preponderance of lifestyle factors linked to poorer physical health, such as higher rates of sedentary behaviour (Stubbs et al., 2016), unhealthy diet (Aucoin et al., 2020), substance use (Myles et al., 2016) and smoking (Myles et al., 2012). In addition, there is evidence of a shared familial risk between psychotic disorders and metabolic disorders, specifically type 2 diabetes (Foley et al., 2016), indicating that the higher rates of diabetes in those with a psychotic disorder are not entirely attributable to antipsychotic medication. Therefore, while metabolic side‐effects are associated with second‐generation antipsychotic medications (SGAs), it has not yet been possible to disentangle the effects of medication from these other factors.

This distinction is essential for several reasons. First, individuals and clinicians often attribute all of the weight gain and metabolic consequences to the commencement of antipsychotic medication, which can lead to non‐adherence and then subsequent relapse (Haddad et al., 2014). Second, if multiple factors cause the resultant weight gain, it is likely that a range of strategic and targeted interventions will be required to adequately address the issue. Therefore, knowing the proportion of weight gain and other metabolic side‐effects that are attributable to non‐pharmacological factors could improve motivation to address these factors.

The Staged Treatment and Acceptability Guidelines in Early Psychosis (STAGES) study was a randomized controlled trial of intensive psychosocial treatment plus or minus antipsychotic medication for young people with a FEP, who were not at high risk of self‐harm or aggression (Francey et al., 2020). In this study, antipsychotic medication naïve young people were randomized to receive either a second‐generation antipsychotic medication (either risperidone or paliperidone) or placebo. The study also included a group of control participants who did not have a psychotic disorder, did not receive any interventions and were followed up after 6 months. Therefore, this study offers the opportunity to isolate the effects of antipsychotic medication on weight gain and other metabolic factors from lifestyle and disorder‐related factors in a randomized design.

There were multiple aims to the current study. First, we aimed to determine whether there were differences in the anthropometric measurements (weight, waist, resting heart rate and blood pressure) between the FEP placebo group and control participants after 6 months, as any difference would not be as a result of medication and would be attributable to illness related factors, such as negative symptoms, lifestyle factors (sedentary behaviour, diet) or genetics. Second, we aimed to determine whether there was a difference in the anthropometric measurements between the FEP medication group and FEP placebo group after three and 6 months. Third, we aimed to determine whether there were differences in the fasting glucose and lipid profile between the FEP medication group and FEP placebo group after 3 months. Any differences identified from the second or third aims would be attributable to the specific effects of antipsychotic medication. There were multiple aims to this study and it was utilizing secondary data and hence, the analysis was exploratory in nature.

## 2 METHODS

### 2.1 Study design

The study was a triple‐blind randomized placebo‐controlled trial (RCT) comparing two groups, who both received intensive psychosocial interventions. One also received a second‐generation antipsychotic medication (FEP medication group), and the other received placebo (FEP placebo group). Allocation occurred on 1:1 ratio, and randomization was stratified for sex and duration of untreated psychosis (DUP; 0–30 days, 31–90 days and 91–180 days). Participants had a follow‐up assessment after 3 and 6 months, while the control participants had an assessment at baseline and at 6 months. The original study was a non‐inferiority RCT that required 60 participants to complete the 6‐month follow‐up study in order to be adequately powered. It was registered with the Australian and New Zealand Clinical Trials Registry (www.anzctr.org) in November 2007 (ID: ACTRN12607000608460) and the study protocol and baseline characteristics of participants has been published (O'Donoghue et al., 2019).

### 2.2 Setting

We conducted this study at the Early Psychosis Prevention and Intervention Centre (EPPIC; McGorry et al., 1996), a specialist clinic for young people aged between 15 and 24 years of age with a FEP. EPPIC is part of Orygen, a public youth mental health service that serves a catchment area of approximately 1 million in the Western region of metropolitan Melbourne, Australia.

### 2.3 Participants

Young people, aged between 15 and 25 years, presenting with FEP, defined as fulfilling criteria for a DSM‐IV psychotic disorder, including (but not limited to): schizophrenia, schizophreniform disorder, delusional disorder, brief psychotic disorder, major depressive disorder with psychotic symptoms, substance induced psychotic disorder or psychosis not otherwise specified (NOS) were screened for eligibility for the study. Potential participants were also required to meet strict inclusion criteria to minimize any risk to the individuals. These were: the ability to provide informed consent; comprehension of the English language; DUP of less than 6 months; living in stable accommodation; low risk to self or others score of <5 on the Suicidality and Hostility items of the Brief Psychiatric Rating Scale (Overall & Gorham, 1962). The participants were antipsychotic naïve. This was defined as a lifetime exposure to antipsychotic medication equivalent to less than 1750 mg chlorpromazine.

### 2.4 Inclusion criteria for this secondary analysis study

In addition to the inclusion criteria to be eligible for the RCT, this secondary analysis study only included individuals who had completed at least 3 months of the study intervention per protocol. As this study had strict safety measures, a proportion of participants were discontinued from the trial medication and may have been commenced on open label antipsychotic medication. These individuals were therefore not included in this analysis, as we only included individuals who received treatment per protocol according to randomization for a period of 6 months for the first objective and for the relevant analysis for the second objective. We only included individuals who had completed 3 months of the intervention per protocol for the relevant analysis for the second objective and for the third objective.

### 2.5 Antipsychotic medication

The antipsychotic medication was either risperidone or paliperidone depending on the period in which the participant was enrolled. The starting dose was 1 mg for risperidone or 3 mg for paliperidone and the study doctor increased the dose of the medication (or placebo) according to the clinical response. The study commenced recruitment in 2008 using risperidone 1 mg tablets and matched placebo and then paused in August 2009, as the study placebo medication became unavailable. It recommenced in June 2012 using paliperidone 3 mg capsules and matched placebo and paused again in March 2013 when the paliperidone and matched placebo became unavailable. The final recruitment phase commenced in September 2013 when funding allowed for the manufacture of placebo and over‐encapsulation of risperidone to match the placebo. Dosing was increased by prescription of additional tablets/capsules as required. Thus, at each phase of the study, the placebo group received placebo tablets or capsules that were identical in appearance, taste and packaging to the active medication. Unless the study medication was discontinued (see below safety measures), participants were not prescribed other antipsychotic medication or mood stabilizers during the intervention period.

### 2.6 Instruments

The Structured Clinical Interview for DSM‐IV Axis I disorders (SCID‐I) was used to determine diagnoses (First et al., 1995). Positive psychotic symptoms were assessed using the Expanded Brief Psychiatric Rating Scale (ExBPRS) version 4 and negative symptoms were assessed using the Scale for the Assessment of Negative Symptoms (SANS; Andreasen, 1984). The psychosis subscale of the BPRS consisted of the four individual items: suspiciousness, hallucinations, unusual thought content and conceptual disorganization.

### 2.7 Anthropometric measurements and blood tests

We measured the height, weight, waist circumference, heart rate and blood pressure of participants at baseline and after 3 and 6 months of the clinical trial. For the control participants, we took these measurements at baseline and after 6 months follow‐up. We conducted fasting blood tests at baseline and after 3 and 6 months for the participants of the clinical trial, and this included fasting glucose levels, total cholesterol and triglycerides. The control participants did not undergo blood tests. We used the cut off as a gain of ≥7% of body weight as clinically significant weight gain (ref).

### 2.8 Safety measures

Strict discontinuation criteria were employed to ensure safety, as this study involved the treatment of some participants with FEP with psychosocial interventions alone (plus placebo). Participants had weekly reviews throughout the intervention period. There were five main criteria leading to trial discontinuation: (i) risk to self or others; (ii) worsening of mental state; (iii) failure to improve within 12 weeks of study entry; (iv) at the request of participants or their caregivers and (v) if a participant became pregnant during the intervention period. Risk to self or others was operationally defined by a rating of 5 or more on the BPRS‐4 Suicidality or Hostility subscales maintained for 1 week. Worsening of mental state was operationally defined by a 2‐point increase on the BPRS‐4 positive symptoms subscale of Conceptual Disorganization, Hallucinations, Unusual Thought Content, or Suspiciousness that was maintained for at least 1 week that was not due to substance use, or a decrease in overall functioning, defined by a 20‐point drop in SOFAS score from baseline maintained for 1 month. The failure to satisfactorily recover was operationally defined as a score of 5 or more on the BPRS‐4 Hallucinations, Suspiciousness, and Unusual Thought Content items or a score of 4 or greater on Conceptual Disorganization at the week 12 assessment. Participants who discontinued study medication were followed‐up according to the study assessment protocol and the treatment they received was recorded.

### 2.9 Recruitment of control participants

We recruited healthy young people as controls from advertisements via social media and flyers posted in the local community. We aimed to recruit a representative control group and therefore they were aged between 15 and 25 and had a similar breakdown of sex and socioeconomic status to the participants with FEP.

### 2.10 Management of missing data

The data was inspected for missing data. Over 50% of data was missing in relation to the follow‐up data at 3 months and this was even higher for the 6 months follow‐up assessment point. Therefore, multiple imputation was deemed inappropriate (Andreasen, 1984). We therefore we excluded participants with missing data and emphasizing the high proportion of missing data in the results and discussion of the findings (Jakobsen et al., 2017).

### 2.11 Statistical analysis

Baseline differences between two groups were tested using independent samples *t*‐tests. For dichotomous categorical variables, Chi‐square tests (*χ* ^2^) were used to determine if there was a difference in the observed outcome compared to the expected outcome and when there was 5 or less variables in any group, Fishers exact test was used and reported. Linear regression was used to determine whether group status (e.g., FEP medication vs FEP placebo) was associated with continuous outcomes at 3‐ and 6‐months (e.g., weight) after adjusting for the baseline measure. Where multiple analyses were performed, a Bonferroni correction was applied, in which the level of significance was obtained by divided 0.05 by the number of analyses conducted. Analysis of covariance (ANCOVA) was conducted with the follow‐up physical health variable as the dependent variable and the baseline measure for the specific physical health variable was entered as a covariate in the model and effect sizes were determined from the partial eta squared Statistics were performed using SPSS v.26 (IBM Corporation, 2019).

## 3 RESULTS

### 3.1 Recruitment

**FIGURE 1:**
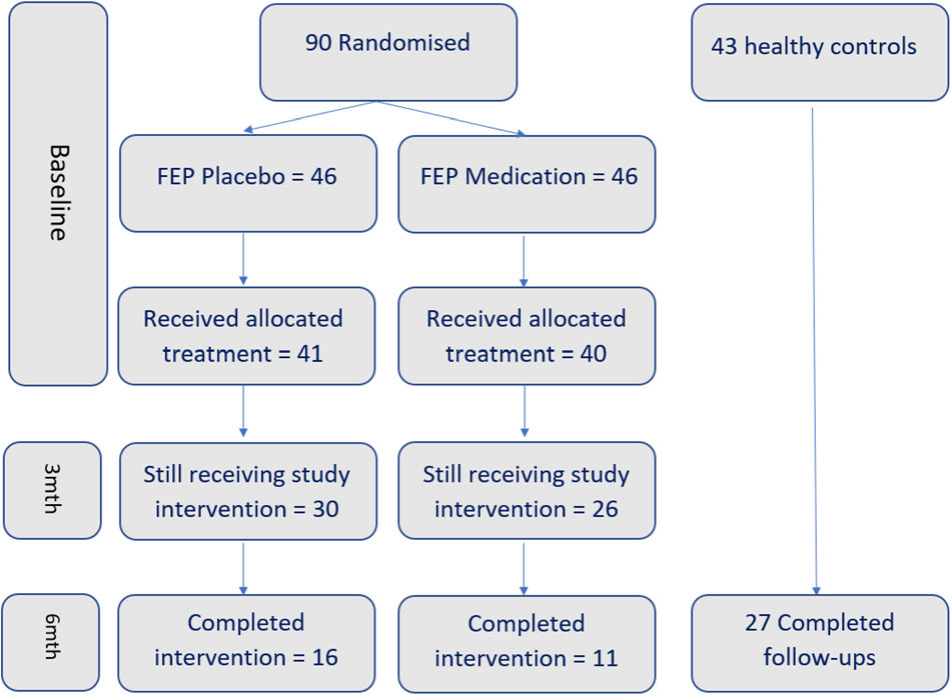
Flow diagram of participants in study

A total of 90 young people were recruited to the trial. Off these, four did not undertake the baseline assessment after providing informed consent and a further five never commenced the study medication, resulting in relevant measures being available for 81 participants. A total of 27 controls were recruited who had an assessment at baseline and after 6 months. A flow diagram of the recruitment and retention of participants and control participants is presented in Figure 1.

### 3.2 Characteristics of participants

The demographic and clinical characteristics of the FEP cohort who had completed the intervention at 3 months and at 6 months and the control participants are presented in Table 1. In summary, of those who completed at least 3 months of the study intervention, 44.6% (*n* = 22) of participants were male, the mean age was 18.3 years (±2.7), 96.4% (*n* = 54) were never married and 73.2% (*n* = 41) were employed in some capacity.

**TABLE 1:** Baseline demographic & clinical features of study participants who completed ≥3 months intervention and healthy controls

	Total FEP cohort *N* = 56	Antipsychotic medication FEP group *N* = 26	Placebo FEP group *N* = 30	Healthy control group *N* = 27
Baseline characteristics	Means (SDs) or percentages (*n*)	Means (SDs) or percentages (*n*)	Means (SDs) or percentages (*n*)	Means (SDs) or percentages (*n*)
*Demographics*				
Age at service entry, years	18.3 (2.7)	18.4 (2.6)	18.2 (2.8)	19.6 (3.5)
Sex: % male	44.6 (25)	46.2 (12)	43.3 (13)	29.6 (8)
Marital status, % never married	96.4 (54)	96.2 (25)	96.7 (29)	85.2 (23)
Work status, %				
*Employed in some capacity*	73.2 (41)	76.9 (20)	70.0 (21)	66.7 (18)
*Student*	8.9 (5)	7.7 (2)	10.0 (3)	0 (0)
*Unemployed/Home duties*	17.9 (10)	15.4 (4)	20.0 (6)	33.3 (9)
Australian‐born, %	91.1 (5)	92.3 (24)	90.0 (27)	74.1 (20)
*Illness and clinical features*				
DSM‐IV Diagnosis, %				
*Schizophrenia/Schizophreniform*	30.4 (17)	26.9 (7)	33.3 (10)	‐
*Affective (Bipolar/Depressive)*	21.4 (12)	26.9 (7)	16.7 (5)	‐
*Schizoaffective*	0 (0)	0 (0)	0 (0)	‐
*Substance induced psychotic disorder*	14.3 (8)	11.5 (3)	16.7 (5)	‐
*delusional/NOS/brief psychosis*	33.9 (19)	34.6 (9)	33.3 (10)	‐
Total psychopathology score (Total BPRS),	56.4 (8.9)	53.7 (7.9)	58.6 (9.2)	31.8 (3.9)
Psychotic symptoms severity (BPRS Psychosis subscale)	13.9 (3.4)	12.9 (3.4)	14.7 (3.3)	4.1 (0.3)
Negative symptoms (SANS)	34.8 (18.2)	32.6 (18.0)	36.7 (18.4)	‐
Social & Occupational Functioning (SOFAS)	58.1 (8.1)	57.3 (7.6)	58.8 (8.7)	81.3 (9.3)

### 3.3 Follow‐up rates and missing data

A total of 69.1% (*n* = 56) of the 81 FEP participants remained on the study medication at the 3‐month follow‐up assessment (*n* = 26 medication group; *n* = 30 placebo group) and 33.3% (*n* = 27) at the 6‐month assessment (*n* = 11 medication group; *n* = 16 placebo group). For those who did not discontinue the study medication by the 3‐month follow‐up, there was follow‐up information for weight for 60.7% (*n* = 34) and there was follow‐up information for weight for 70.3% (*n* = 19) for those who had completed the entire 6‐month study intervention. Comparisons of the demographic and clinical characteristics of those who had data available at the 3‐ and 6‐month follow‐up assessments to those with missing data, for the total cohort and then according to group allocation, are presented in Tables S1–S3. Of note, in the total cohort, participants who had data available for the 6‐month follow‐up had less severe psychotic symptoms (13.1 vs. 15.0, *p* = .030) and in the sub‐group who were allocated to the placebo group, those who had data available at 6 months were less likely to have a diagnosis of schizophrenia compared to those with no data available (0% vs. 50%, *p* = .030).

### 3.4 Aim 1: Changes attributable to non‐medication factors—Comparison of anthropometric measurements between FEP placebo group and control participants after 6 months

The mean changes in anthropometric measurements between the FEP placebo group and the control participant group are presented in Table 2. In summary, the FEP placebo group gained a mean of 2.4 kg (±4.9) after 6 months compared to 1.1 kg (±4.9) in the control participants (*p* = .454). After 6 months, 27.3% (*n* = 3) of the FEP Placebo group gained ≥7% of their body weight compared to 23.1% (*n* = 6) of the control participant group (*χ* ^2^ = .07, *df* = 1, *p* = .786).

**TABLE 2:** Comparison of anthropometric measurements between FEP‐placebo and healthy controls after 6 months

	FEP—placebo		Healthy controls		Difference between groups at 6 months		
	*N*	Mean (SD)	*N*	Mean (SD)	Difference [95% C.I.]	*t* (*df*)	*p*
*Weight (kg)*							
Baseline	11	73.0 (19.5)	26	68.1 (12.6)			
6 months	11	75.5 (20.1)	26	69.1 (13.8)			
Difference		2.4 (4.9)		1.1 (4.9)	1.3 [−2.2, 4.89]	0.76 (35)	.454
*Waist circumference (cm)*							
Baseline	9	82.0 (10.3)	25	80.5 (9.5)			
6 months	9	85.9 (14.9)	25	79.5 (10.5)			
Difference		3.9 (7.1)		−0.9 (4.9)	4.8 [0.44, 9.21]	2.24 (32)	.032
*Systolic blood pressure (mmHg)*							
Baseline	10	121.7 (19.2)	26	116.2 (11.7)			
6 months	10	113.5 (19.2)	26	109.4 (12.6)			
Difference		−8.2 (18.3)		−6.7 (9.9)	−1.5 [−10.5, 7.6]	−0.33 (34)	.743
*Diastolic blood pressure (mmHg)*							
Baseline	10	78.9 (7.3)	26	74.6 (9.4)			
6 months	10	70.8 (8.7)	26	72.3 (8.2)			
Difference		−8.1 (7.7)		−2.3 (7.4)	−5.8 [−11.4, −1.2]	−2.1 (34)	.046
*Heart rate (bpm)*							
Baseline	3	81.0 (13.1)	26	70.9 (13.6)			
6 months	3	66.0 (11.4)	26	69.8 (13.4)			
Difference		−15.0 (14.8)		−1.2 (9.1)	−13.9 [−25.9, −1.8]	−2.4 (27)	.026

*Note*: Significance level was set at .01 following a Bonferroni correction.

Abbreviations: bpm, beats per minute; cm, centimetres; kg, kilograms, mmHg, millimetres change in mercury.

There was a significant difference in the changes in waist circumference after 6 months, with the FEP placebo group gaining 3.9 cm (±7.1) compared to the control participants losing 0.9 cm (±4.9; *p* = .032). There was no significant difference in systolic blood pressure. In the FEP placebo group there was a reduction of 8.2 mmHg (±18.3) in diastolic blood pressure compared to a reduction of 2.3 mmHg (±7.4) in the control participants (*p* = .046)., however this was not significant after adjusting for multiple analyses There was a large amount of missing data in relation to heart rate in the FEP placebo group, which experienced a reduction of 15.0 bpm (±14.8) compared to 1.2 bpm (±9.1) in the control participants (*p* = .026).

### 3.5 Aim 2: Changes attributable to antipsychotic medication ‐ comparison of anthropometric measurements between the FEP medication group and FEP placebo group after 3 and 6 months

The comparison of the anthropometric measurements between FEP medication group and FEP placebo group are presented in Table 3 and the results of the linear regression for each anthropometric factor, controlled for the baseline level, are presented in Table 4. In summary, after 3 months, young people in the FEP medication group gained 3.7 kg (±3.9) compared to 1.4 kg (±5.4) in the FEP placebo group (*p* = .202). After 6 months, those in the FEP Medication group had gained 4.1 kg (±4.5) and the FEP placebo group had gained 2.4 kg (±4.9; *p* = .444). After 3 months, 30.8% (*n* = 4) of those in the FEP medication group gained ≥7% of their body weight compared to 9.5% (*n* = 2) of those in the FEP placebo group (*χ* ^2^ = 2.49, *df* = 1, *p* = .114). After 6 months, 37.5% (*n* = 3) of those in the FEP medication group gained ≥7% of their body weight compared to 27.3% (*n* = 3) of the FEP Placebo group (*χ* ^2^ = .22, *df* = 1, *p* = .636).

**TABLE 3:** Comparison of anthropometric measurements between FEP‐medication group and FEP‐placebo after three and 6 months

	3 month follow‐up							6 month follow‐up						
	FEP—medication		FEP—placebo		Difference between groups			FEP—medication		FEP—placebo		Difference between groups		
	*N*	Mean (SD)	*N*	Mean (SD)	Difference [95% C.I.]	*t* (*df*)	*p*	*N*	Mean (SD)	*N*	Mean (SD)	Difference (95% C.I.)	*t* (*df*)	*p*
*Weight (kg)*														
Baseline	13	70.5 (10.6)	21	71.7 (17.5)				8	72.6 (13.0)	11	73.0 (19.5)			
Follow‐up	13	74.1 (8.9)	21	73.1 (17.4)				8	76.8 (12.4)	11	75.5 (20.1)			
Difference		3.7 (3.9)		1.4 (5.4)	2.3 [−1.3, 5.8]	1.3 (32)	.202		4.1 (4.5)		2.4 (4.9)	1.7 [−2.9, 6.4]	0.8 (17)	.444
*Waist (cm)*														
Baseline	7	82.1 (8.9)	10	83.4 (7.1)				5	87.6 (6.9)	9	89.7 (9.8)			
Follow‐up	7	83.4 (7.1)	10	83.7 (14.2)				5	89.7 (9.8)	9	85.9 (14.9)			
Difference		1.2 (6.4)		1.6 (6.4)	−0.4 [−7.1, 6.3]	−0.1 (15)	.904		2.1 (7.3)		3.9 (7.1)	−1.8 [−10.5, 6.9]	−0.5 (12)	.662
*Systolic bp (mmHg)*														
Baseline	13	123.4 (9.4)	19	119.0 (15.8)				8	122.1 (13.4)	10	121.7 (19.2)			
Follow‐up	13	111.7 (8.1)	19	118.6 (13.6)				8	107.9 (12.1)	10	113.5 (19.2)			
Difference		−11.7 (7.7)		−0.4 (12.2)	−11.3[−19.1,‐3.5]	−3.0 (30)	.006		−14.3 (9.1)		−8.2 (16.3)	−6.1 [−19.8, 7.7]	−0.9 (16)	.364
*Diastolic bp (mmHg)*														
Baseline	13	78.4 (7.2)	19	76.0 (9.0)				8	76.1 (10.8)	10	78.9 (10)			
Follow‐up	13	72.0 (9.2)	19	72.0 (12.5)				8	66.6 (8.8)	10	70.8 (8.7)			
Difference		−6.4 (7.8)		−4.0 (14.4)	−2.4 [−11.3, 6.6]	−0.5 (30)	.591		−9.5 (5.9)		−8.1 (7.7)	−1.4 [−8.4, 5.6]	−0.4 (16)	.677
*Heart rate (bpm)*														
Baseline	7	81.9 (12.7)	9	82.3 (12.0)				3	73.3 (6.4)	3	81.0 (13.1)			
Follow‐up	7	75.3 (9.5)	9	80.6 (13.5)				3	69.3 (10.3)	3	66.0 (11.4)			
Difference		−6.6 (7.6)		−1.8 (12.6)	−4.8 [−16.4, 6.8]	−0.9 (14)	.392		−4.0 (4.0)		−15.0 (14.8)	11.0 [−14.6,35.6]	1.3 (4)	.282

*Note*: Significance level was set at .01 following a Bonferroni correction.

Abbreviations: bpm, beats per minute; cm, centimetres; kg, kilograms, mmHg, millimetres change in mercury.

**TABLE 4:** Analysis of covariance (ANCOVA) for physical health outcome between FEP‐medication and FEP placebo groups at 3 and 6 months follow‐up. Controlled for baseline measurement

	3 months follow‐up				6 months follow‐up			
	*N*	*F* (*df*)	*p*	Effect size	*N*	*F*	*p*	Effect size
*Weight*								
Group	Medication = 13, placebo = 21	1.61 (1,31)	.215	0.05	Medication = 13, placebo = 21	0.58	.459	0.035
Baseline weight		269.08	<.001			200.63	<.001	
*Waist*								
Group	Medication = 7, placebo = 10	0.014 (1,14)	.91	0.001	Medication = 5, placebo = 9	0.58	.462	0.050
Baseline waist		35.21	<.001			31.97	<.001	
*Resting heart rate*								
Group	Medication = 7, placebo = 9	1.00 (1,13)	.335	0.07	Medication = 3, placebo = 3	0.53	.521	0.149
Baseline heart rate		7.52	.017			0.87	.419	
*Systolic Blood Pressure*								
Group	Medication = 13, placebo = 19	8.20 (1,29)	.008	0.22	Medication = 8, placebo = 10	0.96	.343	0.060
Baseline systolic bp		22.29	<.001			10.09	.007	
*Diastolic Blood Pressure*								
Group	Medication = 13, placebo = 19	0.04 (1,29)	.841	0.001	Medication = 8, placebo = 10	0.55	.471	0.035
Baseline diastolic bp		2.15	.154			13.86	.002	

**FIGURE 2:**
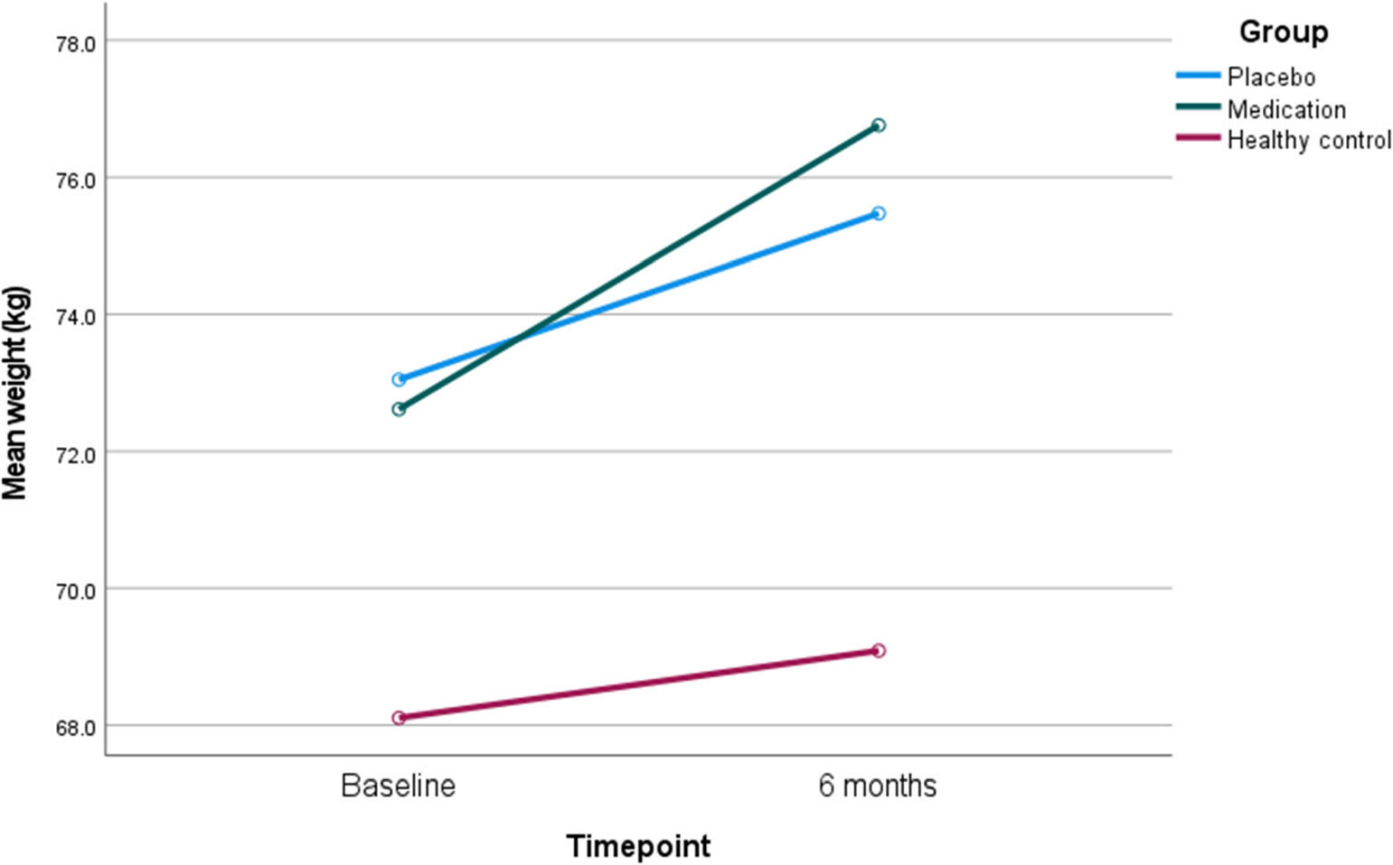
Changes in weight after 6 months in the participants who completed the 6‐month intervention in the medication and placebo groups and the control group

In relation to waist measurement, there were no significant differences between the FEP medication group and FEP placebo group after 3 (*p* = .904) or 6 months (*p* = .662). After 3 months, the FEP medication group experienced a reduction of 11.3 mmHg 95% C.I. [−19.1, −3.5] in systolic blood pressure in comparison to the FEP placebo group (*p* = .006), however there was no difference in diastolic blood pressure between groups at either follow‐up. There was no difference in resting heart rate between the two groups at either follow‐up point. The above findings remained consistent when the outcomes were assessed using ANCOVA controlling for the baseline measurement of the physical health measure, as displayed in Table 4. The changes in weight from baseline to 6‐month assessment in the medication, placebo and control group are presented as plots in Figure 2 and the remainder of the anthropometric measures and fasting glucose and lipids are presented in Supporting Information.

### 3.6 Aim 3: Changes attributable to antipsychotic medication: Comparison of the fasting haematological tests between the FEP medication group and FEP placebo group after 3 months

There were no significant differences between the baseline and 3‐month outcomes for the FEP medication group or the FEP placebo group in relation to fasting blood glucose (*p* = .62), fasting triglycerides (*p* = .70) or fasting cholesterol (*p* = 0.49). The baseline and 3‐month outcomes for these factors are presented in Table 5.

**TABLE 5:** Comparison of fasting blood tests measurements between FEP‐medication and FEP pPlacebo after 3 months

	FEP—Medication		FEP‐placebo		Difference between groups at 3 months		
	*N*	Mean (SD)	*N*	Mean (SD)	Difference [95% C.I.]	*t* (*df*)	*p*
*Fasting glucose (mmol/L)*							
Baseline	6	4.88 (0.24)	5	4.80 (0.35)	−0.11 [−0.59, 0.37]	−0.51 (9)	.620
3 months	6	4.63 (0.38)	5	4.66 (0.48)			
Difference		−0.25 (0.44)		−0.14 (0.19)			
*Fasting triglycerides (mmol/L)*							
Baseline	5	1.20 (0.60)	4	0.85 (0.17)	0.14 [−0.68, 0.96]	0.40 (7)	.699
3 months	5	1.24 (0.81)	4	0.75 (0.17)			
Difference		0.04 (0.62)		−0.10 (0.34)			
*Fasting cholesterol (mmol/L)*							
Baseline	5	4.96 (1.74)	4	4.55 (0.79)	−0.36 [−1.50, 0.79]	−0.74 (7)	.485
3 months	5	4.78 (1.19)	4	4.73 (0.55)			
Difference		−0.18 (0.82)		0.18 (0.56)			

## 4 DISCUSSION

### 4.1 Summary of findings

Over the course of the first 6 months after presentation with a FEP, there were no significant changes in weight, waist circumference, heart rate or blood pressure that could be attributed to non‐medication factors. The FEP placebo group experienced more weight gain, on average, than the control participants, but this difference was not statistically significant. Similarly, when comparing the FEP Medication and FEP placebo group, there was an absolute difference in weight that could be attributable to antipsychotic medication, but this difference was not statistically significant. There was a reduction in systolic blood pressure after 3 months that was statistically significant, that could be attributable to antipsychotic medication, however there were no statistical differences in relation to waist circumference, resting heart rate or fasting lipids or glucose.

### 4.2 Strengths and Limitations

The findings of this study need to be considered in the context of several factors. The most important limitation is the sample size, resulting in low power for detecting statistically significant differences between groups. This small sample size resulted from the very narrow eligibility criteria for the study and the high attrition due to participants either discontinuing the study medication, having missing data at follow‐up, or the participant meeting the strict discontinuation criteria, which were necessary to ensure the safe running of the trial. Therefore, even considering the small sample size. Lack of statistical power and wide confidence intervals for any findings, this is the first study to compare the physical health outcomes between young people with an FEP receiving either antipsychotic medication or placebo over a period of 6 months. There are other potential limitations to the study that might introduce bias. First, side‐effects were one of the reasons why young people discontinued the study medication. Therefore, the young people who experienced weight gain or other metabolic disturbance may have elected to discontinue the study medication, thereby introducing a bias to include participants who experienced less weight gain. This potential bias would have resulted in an under‐estimation of the true weight gain associated with antipsychotic medication. In addition, we did not account for non‐compliance in this article study and any non‐compliance in the FEP‐medication group would have led to an under‐estimation of the true metabolic side‐effects of the medication while non‐compliance in the placebo group would not have any impact. We also elected to conduct Bonferroni corrections where applicable; however, the study consisted of secondary analysis from an RCT and was exploratory in nature. It could be argued that these corrections were unnecessary and while the aim of controlling for multiple analysis was to reduce type I errors, it may have led to type II errors (Feise, 2002). Therefore, we have provided the full *p* values in Tables 2, 3, 4, 5, so that the reader can interpret with the results with or without a Bonferroni correction.

### 4.3 Clinical implications

The findings of this study suggest that the metabolic comorbidities associated with psychotic disorders are not entirely consequences of antipsychotic medication. The aetiology of the metabolic complications are multifactorial, and as a result the interventions aimed at preventing or reducing these metabolic consequences should also be multifaceted. Lifestyle interventions, aimed at improving dietary intake and levels of physical activity should be provided to all individuals presenting with FEP. These lifestyle interventions can be effective in preventing the weight gain that has been attributed to antipsychotic medication (Curtis et al., 2016), although our findings indicate that some of this weight gain and metabolic side‐effects could be attributable to non‐medication factors. Based on data from mood disorder research, such interventions may also have benefits on core symptomatology, and establishing this in psychosis is a research priority. However, some individuals may require more targeted interventions, such as those who smoke tobacco or use illicit substances. Furthermore, antipsychotic medications with a lower propensity for weight gain and metabolic complications should be used as first line agents (Leucht et al., 2013; Nguyen et al., 2020). If these interventions are not effective in attenuating the development of metabolic complications, then the use of metformin, as a preventative agent, is also indicated (Correll et al., 2013).

In line with the early intervention philosophy, prevention should be the target. Individuals who are identified as being at ultra‐high risk for psychosis (UHR) have lower physical activity than their healthy peers, they consume more calories and have higher rates of smoking and alcohol intake (Carney et al., 2016). However, their BMIs do not differ from control participants and the averages for UHR young people lie within the healthy range (Carney et al., 2016). Therefore, in the UHR stage, risk factors for metabolic complications are present but have not yet developed into physical comorbidities. This is in keeping with the findings in a systematic review that at the time of presentation, there are no differences in the metabolic health of young people with FEP compared to healthy peers (Foley & Morley, 2011), although since this review, individual studies have demonstrated that those with a FEP have higher waist‐to‐hip ratios (Shah et al., 2019), are more likely to be overweight or obese (Kolenic et al., 2018); and have higher levels of insulin resistance, total and low‐density lipoprotein cholesterol, and triglycerides (Keinänen et al., 2018). These findings emphasize that the ideal time to implement physical health interventions is either at the UHR stage or immediately at the time of presentation with a first episode of psychosis.

### 4.4 Conclusions

While limited by small numbers and high attrition, the findings of this study suggest that some of the metabolic complications observed in psychotic disorders could be attributable to factors other than medication. These findings emphasize the need to deliver physical health interventions either at the ultra‐high risk for psychosis stage if feasible or immediately at the time of presentation with a FEP.

## Supporting information


**FIGURE S1:** Changes in waist circumference in the medication, placebo and control groups from baseline to six months
**FIGURE S2**: Changes in resting heart rate in the medication, placebo and control groups from baseline to six months
**FIGURE S3**: Changes in systolic blood pressure in the medication, placebo and control groups from baseline to six months
**FIGURE S4**: Changes in diastolic blood pressure in the medication, placebo and control groups from baseline to six months
**FIGURE S5**: Changes in fasting glucose levels in the medication and placebo groups between baseline, three months and six months
**FIGURE S6**: Changes in fasting triglyceride levels in the medication and placebo groups between baseline, three months and six months
**FIGURE S7**: Changes in fasting cholesterol levels in the medication and placebo groups between baseline, three months and six months

**TABLE S1:** Total cohort comparison of the demographic, clinical and physical health characteristics of those who remained on study medication and had data available at 3 months and 6 months compared to those who either discontinued study medication or did not have follow‐up data available
**TABLE S2**: Antipsychotic medication cohort: Comparison of the demographic, clinical and physical health characteristics of those who remained on study medication and had data available at 3 months and 6 months compared to those who either discontinued study medication or did not have follow‐up data available
**TABLE S3**: Placebo cohort: Comparison of the demographic, clinical and physical health characteristics of those who remained on study medication and had data available at 3 months and 6 months compared to those who either discontinued study medication or did not have follow‐up data available

## Data Availability

Ethical approval was not granted for the data to be publicly available, however the corresponding author can be contacted about any queries in relation to the data.
