# Supplementary material for: Isolating the impact of antipsychotic medication on metabolic health: Secondary analysis of a randomized controlled trial of antipsychotic medication versus placebo in antipsychotic medication naïve first‐episode psychosis (the STAGES study)
Source: Early Interv Psychiatry. 2022 Oct 4;17(6):597–607. doi: 10.1111/eip.13353 (PMC10947230; doi:10.1111/eip.13353)
Supplement: Supplementary file 1 — FIGURE S1: Changes in waist circumference in the medication, placebo and control groups from baseline to six months FIGURE S2: Changes in resting heart rate in the medication, placebo and control groups from baseline to six months FIGURE S3: Changes in systolic blood pressure in the medication, placebo and control groups from baseline to six months FIGURE S4: Changes in diastolic blood pressure in the medication, placebo and control groups from baseline to six months FIGURE S5: Changes in fasting glucose levels in the medication and placebo groups between baseline, three months and six months FIGURE S6: Changes in fasting triglyceride levels in the medication and placebo groups between baseline, three months and six months FIGURE S7: Changes in fasting cholesterol levels in the medication and placebo groups between baseline, three months and six months [file EIP-17-597-s001.docx]

**Changes in anthropometric measures from baseline to six months in those allocated to medication or placebo group and who completed the six-month study intervention and healthy controls (supplementary figures 1-4).**


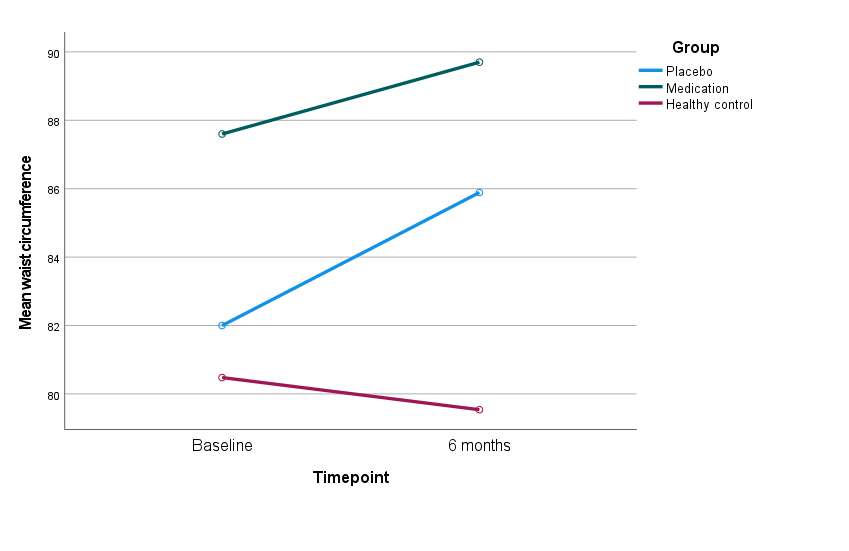


*Supplementary Figure 1: Changes in waist circumference in the medication, placebo and control groups from baseline to six months*


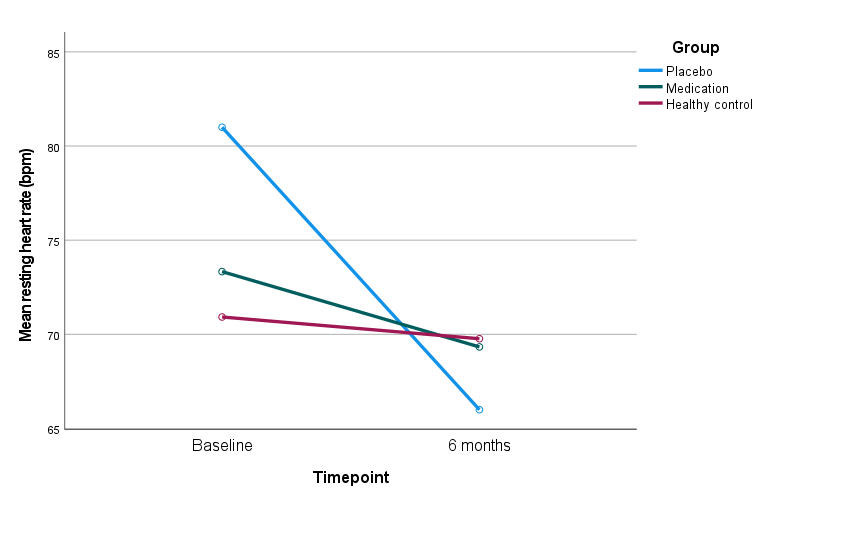


*Supplementary Figure 2: Changes in resting heart rate in the medication, placebo and control groups from baseline to six months*


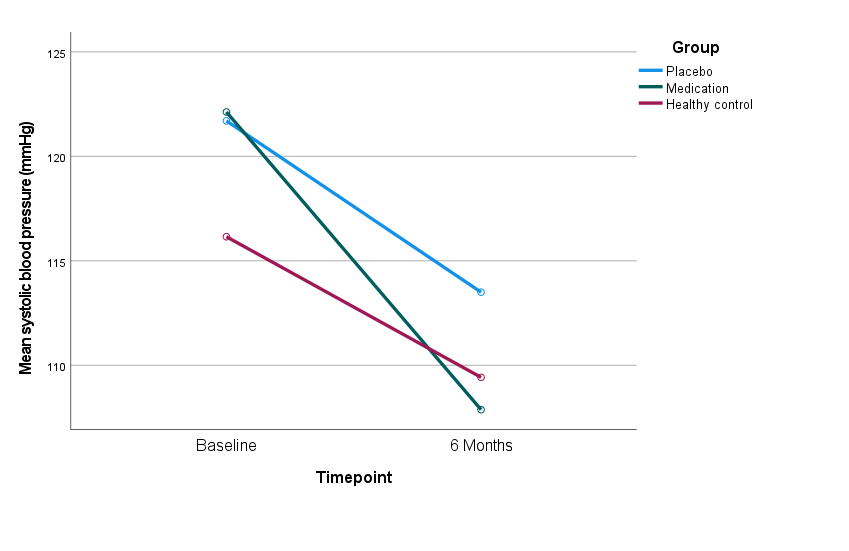


*Supplementary Figure 3: Changes in systolic blood pressure in the medication, placebo and control groups from baseline to six months*


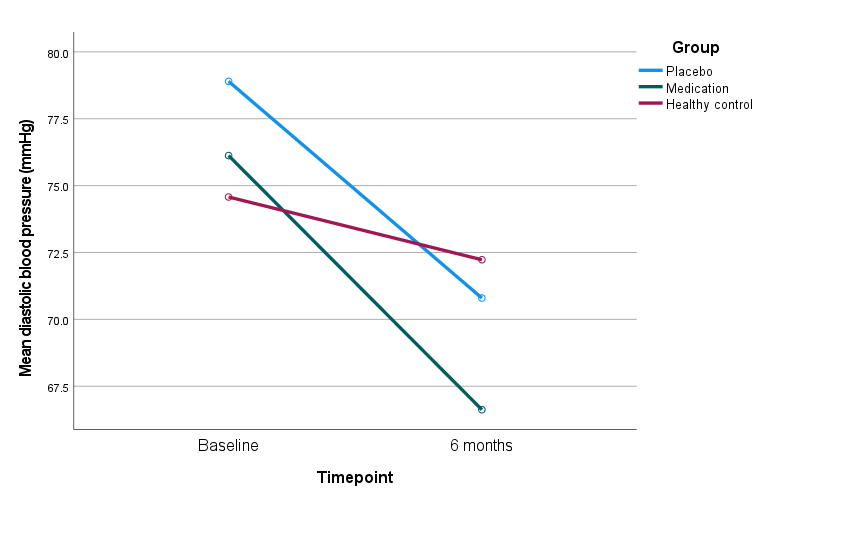


*Supplementary Figure 4: Changes in diastolic blood pressure in the medication, placebo and control groups from baseline to six months*

**Changes in fasting glucose and lipids from baseline to six months in those allocated to medication or placebo group and who completed the six-month study intervention (supplementary figures 5-7).**


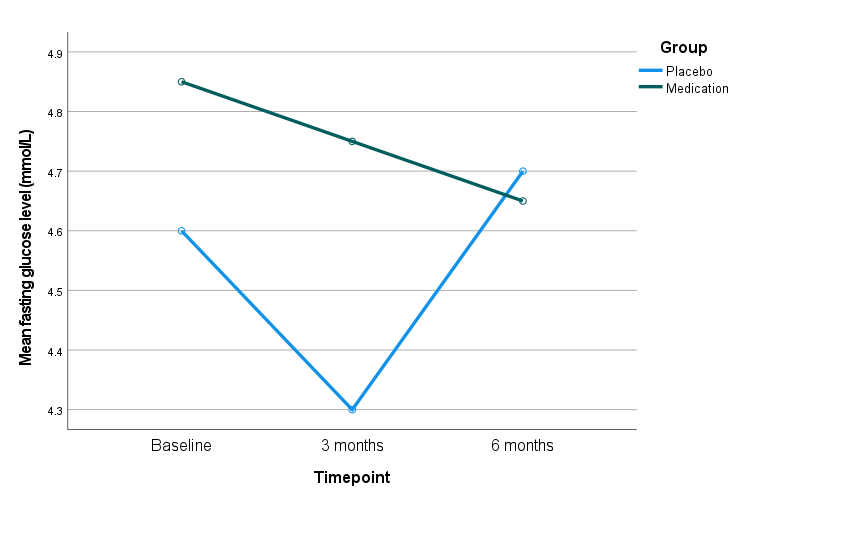


*Supplementary Figure 5: Changes in fasting glucose levels in the medication and placebo groups between baseline, three months and six months*

*
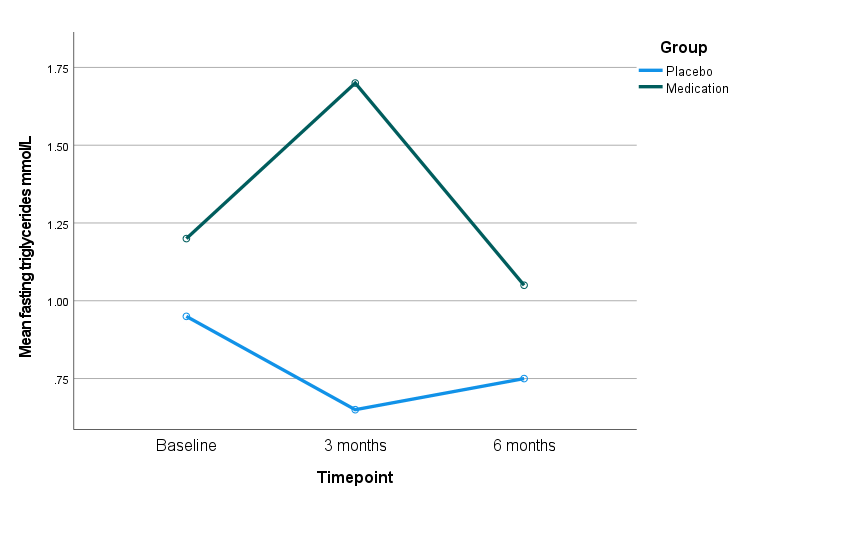
*

*Supplementary Figure 6: Changes in fasting triglyceride levels in the medication and placebo groups between baseline, three months and six months*


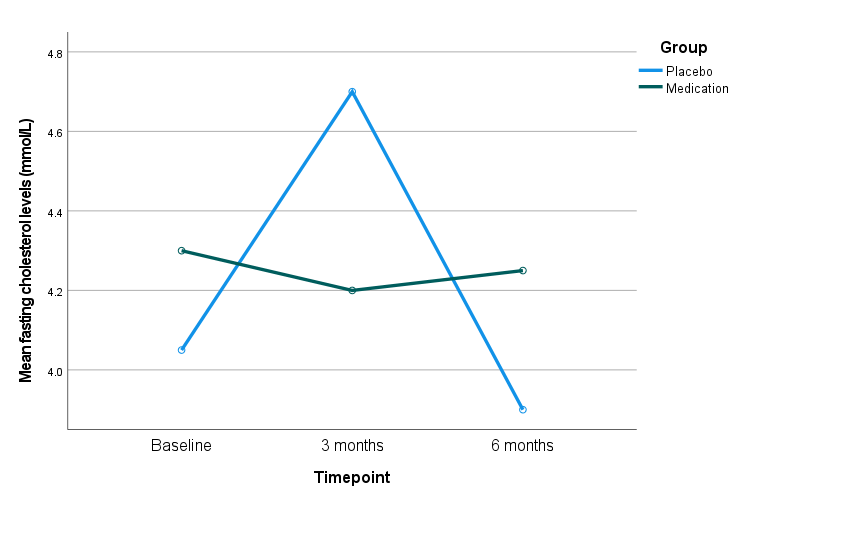


*Supplementary Figure 7: Changes in fasting cholesterol levels in the medication and placebo groups between baseline, three months and six months*
