# Supplementary material for: Isolating the impact of antipsychotic medication on metabolic health: Secondary analysis of a randomized controlled trial of antipsychotic medication versus placebo in antipsychotic medication naïve first‐episode psychosis (the STAGES study)
Source: Early Interv Psychiatry. 2022 Oct 4;17(6):597–607. doi: 10.1111/eip.13353 (PMC10947230; doi:10.1111/eip.13353)
Supplement: Supplementary file 2 — TABLE S1: Total cohort comparison of the demographic, clinical and physical health characteristics of those who remained on study medication and had data available at 3 months and 6 months compared to those who either discontinued study medication or did not have follow‐up data available TABLE S2: Antipsychotic medication cohort: Comparison of the demographic, clinical and physical health characteristics of those who remained on study medication and had data available at 3 months and 6 months compared to those who either discontinued study medication or did not have follow‐up data available TABLE S3: Placebo cohort: Comparison of the demographic, clinical and physical health characteristics of those who remained on study medication and had data available at 3 months and 6 months compared to those who either discontinued study medication or did not have follow‐up data available [file EIP-17-597-s002.docx]

| ***Supplementary Table 1: Total cohort comparison of the demographic, clinical and physical health characteristics of those who remained on study medication and had data available at 3 months and 6 months compared to those who either discontinued study medication or did not have follow-up data available*** | | | | | | | | | | | | | | | | | | | | | | | | |  |  |
| --- | --- | --- | --- | --- | --- | --- | --- | --- | --- | --- | --- | --- | --- | --- | --- | --- | --- | --- | --- | --- | --- | --- | --- | --- | --- | --- |
|  | 3 months | | | | | | | | | | | | 6 months | | | | | | | | | | | |  |  |
|  | Followed up  (N=34) | | | Not followed up (N=47) | | | Statistical test of difference | | | p | | | Followed up  (N=19) | | | Not followed up (N=62) | | | Statistical test of difference | | | p | | |  |  |
| **Demographic** | median (I.Q.R.) | | | median (I.Q.R.) | | | Z | | |  | | | median (I.Q.R.) | | | median (I.Q.R.) | | | Z | | |  | | |  |  |
| Age | 18.0  (16.0 – 21.0) | | | 18.0  (16.0 – 20.0) | | | -0.19 | | | .852 | | | 19.0  (16.0 – 20.0) | | | 18.0  (16.0 – 20.5) | | | -0.50 | | | .620 | | |  |  |
|  | % (n) | | | % (n) | | | X^2^ (df) | | |  | | | % (n) | | | % (n) | | |  | | |  | | |  |  |
| Sex, % male | 52.9 (18) | | | 38.3 (18) | | | 1.71 (1) | | | .191 | | | 52.6 (10) | | | 41.9 (26) | | | 0.67 (1) | | | .412 | | |  |  |
| Marital status, % never married | 94.1 (32) | | | 97.9 (46) | | | 3.52 (3) | | | .319 | | | 100 (19) | | | 95.2 (59) | | | 0.96 (3) | | | .812 | | |  |  |
| Work status, % employed | 67.6 (23) | | | 78.7 (37) | | | 1.42 (2) | | | .492 | | | 68.4 (13) | | | 75.8 (47) | | | 4.02 (2) | | | .134 | | |  |  |
| Migrant status, % Australian | 2.9 (1) | | | 13.0 (6) | | | 2.50 (1) | | | .114 | | | 0 (0) | | | 11.5 (7) | | | 2.39 (1) | | | .122 | | |  |  |
| **Clinical** |  | | |  | | |  | | |  | | |  | | |  | | |  | | |  | | |  |  |
| Diagnosis, % schizophrenia spectrum | 29.4 (10) | | | 40.4 (19) | | | 1.04 (1) | | | .308 | | | 15.8 (3) | | | 41.9 (26) | | | 4.32 (1) | | | .055 | | |  |  |
|  | mean (sd) | | | mean (sd) | | | t-test (df) | | |  | | | mean (sd) | | | mean (sd) | | |  | | |  | | |  |  |
| Total BPRS | 57.7 (9.2) | | | 58.1 (9.5) | | | -0.20 (78) | | | .839 | | | 54.8 (8.0) | | | 58.9 (9.5) | | | -1.67 (78) | | | .099 | | |  |  |
| BPRS Psychosis | 13.9 (3.5) | | | 15.0 (3.2) | | | -1.45 (78) | | | .152 | | | 13.1 (3.1) | | | 15.0 (3.3) | | | -2.21 (78) | | | .030 | | |  |  |
| SANS | 39.6 (17.7) | | | 32.7 (18.6) | | | 1.67 (78) | | | .098 | | | 34.9 (19.5) | | | 35.8 (18.3) | | | -0.18 (78) | | | .856 | | |  |  |
| SOFAS | 57.3 (8.6) | | | 56.0 (9.9) | | | 0.48 (43) | | | .637 | | | 54.5 (7.5) | | | 57.5 (9.6) | | | -0.98 (43) | | | .166 | | |  |  |
| **Physical health** |  | | |  | | |  | | |  | | |  | | |  | | |  | | |  | | |  |  |
| Weight | 71.2 (15.0) | | | 72.0 (19.7) | | | -0.19 (67) | | | .849 | | | 72.9 (16.7) | | | 71.1 (17.9) | | | 0.37 (67) | | | .715 | | |  |  |
| Waist | 82.0 (9.9) | | | 85.4 (15.5) | | | -0.81 (35) | | | .421 | | | 83.7 (9.1) | | | 83.0 (14.2) | | | 0.14 (35) | | | .890 | | |  |  |
| Systolic BP | 119.9 (14.3) | | | 120.5 (14.9) | | | -0.16 (63) | | | .871 | | | 123.1 (16.8) | | | 119.0 (13.5) | | | 1.03 (63) | | | .306 | | |  |  |
| Diastolic BP | 76.4 (8.7) | | | 77.2 (11.5) | | | -0.30 (63) | | | .763 | | | 78.6 (9.5) | | | 76.1 (10.3) | | | 0.91 (63) | | | .364 | | |  |  |
| Heart rate | 79.4 (11.4) | | | 78.0 (11.9) | | | 0.421 (48) | | | .764 | | | 81.5 (11.2) | | | 77.8 (11.6) | | | 1.03 (48) | | | .316 | | |  |  |
| Fasting glucose | 4.7 (0.3) | | | 5.2 (1.6) | | | -1.39 (45) | | | .171 | | | 4.8 (0.4) | | | 5.1 (1.5) | | | -0.54 (45) | | | .595 | | |  |  |
| Fasting triglycerides | 1.0 (0.5) | | | 0.9 (0.3) | | | 1.22 (43) | | | .230 | | | 1.0 (0.4) | | | 0.9 (0.4) | | | 0.20 (43) | | | .840 | | |  |  |
| Fasting cholesterol | 4.4 (1.0) | | | 4.0 (0.8) | | | 1.55 (43) | | | .132 | | | 4.5 (1.2) | | | 4.0 (0.8) | | | 1.64 (43) | | | .108 | | |  |  |
| ***Supplementary Table 2: Antipsychotic medication cohort: Comparison of the demographic, clinical and physical health characteristics of those who remained on study medication and had data available at 3 months and 6 months compared to those who either discontinued study medication or did not have follow-up data available*** | | | | | | | | | | | | | | | | | | | | | | | | | | |
|  | | | 3 months | | | | | | | | | | | | 6 months | | | | | | | | | | | |
|  | | | Followed up  (N=13) | | | Not followed up (N= 27) | | | Statistical test of difference | | | p | | | Followed up  (N= 8) | | | Not followed up (N=32) | | | Statistical test of difference | | | p | | |
| **Demographic** | | | median  (I.Q.R.) | | | median (I.Q.R.) | | | Z | | |  | | | median (I.Q.R.) | | | median (I.Q.R.) | | | Z | | |  | | |
| Age | | | 19.0  (16.0 – 20.5) | | | 19.0  (16.0 – 21.0) | | | -0.12 | | | .918 | | | 20.0  (17.0 – 20.0) | | | 18.0  (16.0 – 21.0) | | | -0.69 | | | .505 | | |
|  | | | % (n) | | | % (n) | | | X^2^ (df) | | |  | | | % (n) | | | % (n) | | |  | | |  | | |
| Sex, % male | | | 53.8 (7) | | | 40.7 (11) | | | 0.61 (1) | | | .435 | | | 62.5 (5) | | | 40.6 (13) | | | 1.24 (1) | | | .430 | | |
| Marital status, % never married | | | 92.3 (12) | | | 100 (27) | | | 2.13 (1) | | | .144 | | | 100 (8) | | | 96.9 (31) | | | 0.26 (1) | | | .999 | | |
| Work status, % employed | | | 69.2 (9) | | | 74.1 (20) | | | 0.31 (2) | | | .856 | | | 100 (8) | | | 65.6 (21) | | | 3.79 (2) | | | .150 | | |
| Migrant status, % Australian | | | 0 (0) | | | 15.4 (4) | | | 2.23 (1) | | | .281 | | | 0 (0) | | | 12.9 (4) | | | 1.15 (1) | | | .284 | | |
| **Clinical** | | |  | | |  | | |  | | |  | | |  | | |  | | |  | | |  | | |
| Diagnosis, % schizophrenia spectrum | | | 30.8 (4) | | | 37.0 (10) | | | 0.15 (1) | | | .999 | | | 37.5 (3) | | | 34.4 (11) | | | 0.03 (1) | | | .868 | | |
|  | | | mean (sd) | | | mean (sd) | | | t-test (df) | | |  | | | mean (sd) | | | mean (sd) | | |  | | |  | | |
| Total BPRS | | | 54.3 (7.4) | | | 58.8 (10.7) | | | -1.37 (37) | | | .178 | | | 51.8 (5.2) | | | 58.7 (10.3) | | | -1.86 (37) | | | .071 | | |
| BPRS Psychosis | | | 12.8 (3.3) | | | 14.5 (3.7) | | | -1.35 (37) | | | .185 | | | 12.6 (3.3) | | | 14.3 (3.7) | | | -1.16 (37) | | | .254 | | |
| SANS | | | 35.9 (16.1) | | | 33.5 (19.1) | | | 0.39 (37) | | | .698 | | | 32.1 (21.0) | | | 34.9 (17.5) | | | -0.38 (37) | | | .706 | | |
| SOFAS | | | 55.0 (7.8) | | | 55.5 (10.9) | | | -0.12 (22) | | | .902 | | | 54.0 (7.4) | | | 55.6 (10.2) | | | -0.33 (22) | | | .741 | | |
| **Physical health** | | |  | | |  | | |  | | |  | | |  | | |  | | |  | | |  | | |
| Weight | | | 70.5 (10.6) | | | 71.3 (20.2) | | | -0.14 (32) | | | .891 | | | 72.6 (13.0) | | | 70.5 (18.2) | | | 0.31 (32) | | | .759 | | |
| Waist | | | 82.8 (8.4) | | | 89.2 (14.7) | | | 1.14 (16) | | | .271 | | | 86.2 (7.1) | | | 85.9 (14.3) | | | 0.04 (16) | | | .969 | | |
| Systolic BP | | | 123.4 (9.4) | | | 117 (16.8) | | | 1.10 (28) | | | .279 | | | 122.1 (13.4) | | | 119.4 (14.7) | | | 0.46 (28) | | | .651 | | |
| Diastolic BP | | | 78.4 (7.2) | | | 74.5 (11.2) | | | 1.08 (28) | | | .289 | | | 76.1 (10.8) | | | 76.2 (9.6) | | | -0.03 (28) | | | .980 | | |
| Heart rate | | | 78.3 (10.9) | | | 78.3 (11.5) | | | .01 (21) | | | .990 | | | 76.7 (12.3) | | | 78.9 (10.8) | | | -0.42 (21) | | | .680 | | |
| Fasting glucose | | | 4.8 (0.25) | | | 5.5 (2.1) | | | -0.98 (21) | | | .338 | | | 5.0 (0.3) | | | 5.4 (2.1) | | | -0.50 (21) | | | .622 | | |
| Fasting triglycerides | | | 1.1 (0.5) | | | 0.9 (0.4) | | | 1.06 (20) | | | .303 | | | 1.1 (0.5) | | | 0.9 (0.4) | | | 0.84 (20) | | | .412 | | |
| Fasting Cholesterol | | | 4.7 (1.4) | | | 3.8 (0.8) | | | 1.99 (20) | | | .060 | | | 4.5 (1.6) | | | 3.9 (0.8) | | | 1.14 (20) | | | .268 | | |
| ***Supplementary Table 3: Placebo cohort: Comparison of the demographic, clinical and physical health characteristics of those who remained on study medication and had data available at 3 months and 6 months compared to those who either discontinued study medication or did not have follow-up data available*** | | | | | | | | | | | | | | | | | | | | | | | | | |  |
|  | | 3 months | | | | | | | | | | | | 6 months | | | | | | | | | | | |  |
|  | | Followed up  (N=21) | | | Not followed up (N=20) | | | Statistical test of difference | | | p | | | Followed up (N=11) | | | Not followed up (N=30) | | | Statistical test of difference | | | p | | |  |
| **Demographic** | | Median (I.Q.R.) | | | Median (I.Q.R.) | | |  | | |  | | | Median (I.Q.R.) | | | Median (I.Q.R.) | | |  | | |  | | |  |
| Age | | 18.0  (15.5 – 21.5) | | | 17.5  (16.0 – 19.0) | | | -0.40 | | | .693 | | | 18.0  (15.0 – 21.0) | | | 18.0  (16.0 – 20.0) | | | -0.05 | | | .965 | | |  |
|  | | % (n) | | | % (n) | | |  | | |  | | | % (n) | | | % (n) | | |  | | |  | | |  |
| Sex, % male | | 52.4 (11) | | | 35.0 (7) | | | 1.26 (1) | | | .262 | | | 45.5 (5) | | | 43.3 (13) | | | 0.02 (1) | | | .903 | | |  |
| Marital status, % never married | | 95.2 (20) | | | 95.0 (19) | | | 2.00 (2) | | | .367 | | | 100 (11) | | | 93.3 (28) | | | 0.77 (2) | | | .680 | | |  |
| Work status, % employed | | 66.7 (14) | | | 85.0 (17) | | | 1.89 (2) | | | .389 | | | 45.5 (5) | | | 86.7 (26) | | | 10.9 (2) | | | .004 | | |  |
| Migrant status, % Australian | | 4.8 (1) | | | 10.0 (2) | | | 0.41 (1) | | | .606 | | | 0 (0) | | | 10.0 (3) | | | 1.19 (1) | | | .276 | | |  |
| **Clinical** | |  | | |  | | |  | | |  | | |  | | |  | | |  | | |  | | |  |
| Diagnosis, % schizophrenia spectrum | | 28.6 (6) | | | 45.0 (9) | | | 1.19 (1) | | | .275 | | | 0 (0) | | | 50.0 (15) | | | 8.67 (1) | | | .003 | | |  |
|  | | mean (sd) | | | mean (sd) | | |  | | |  | | | mean (sd) | | | mean (sd) | | |  | | |  | | |  |
| Total BPRS | | 59.8 (9.7) | | | 57. 2(8.0) | | | 0.94 (39) | | | .353 | | | 57.1 (9.1) | | | 59.0 (8.9) | | | -0.61 (39) | | | .549 | | |  |
| BPRS Psychosis | | 14.5 (3.5) | | | 15.6 (2.4) | | | -1.13 (39) | | | .264 | | | 13.4 (3.1) | | | 15.7 (2.8) | | | -2.25 (39) | | | .030 | | |  |
| SANS | | 41.9 (18.6) | | | 31.7 (18.3) | | | 1.77 (39) | | | .085 | | | 37.0 (19.1) | | | 36.8 (19.2) | | | 0.03 (39) | | | .980 | | |  |
| SOFAS | | 58.7 (9.1) | | | 57.2 (7.7) | | | 0.33 (19) | | | .745 | | | 54.9 (8.1) | | | 60.1 (8.6) | | | -1.34 (19) | | | .197 | | |  |
| **Physical health** | |  | | |  | | |  | | |  | | |  | | |  | | |  | | |  | | |  |
| Weight | | 71.7 (17.5) | | | 73.1 (19.7) | | | -0.22 (33) | | | .824 | | | 73.0 (19.5) | | | 71.9 (17.9) | | | 0.18 (33) | | | .859 | | |  |
| Waist | | 81.6 (11.0) | | | 78.6 (11.0) | | | 0.46 (17) | | | .651 | | | 82.0 (10.3) | | | 79.7 (14.0) | | | 0.40 (17) | | | .691 | | |  |
| Systolic BP | | 117.6 (16.6) | | | 123.7 (12.0) | | | -1.20 (33) | | | .241 | | | 123.7 (19.4) | | | 118.6 (12.6) | | | 0.94 (33) | | | .352 | | |  |
| Diastolic BP | | 75.2 (9.5) | | | 80.2 (11.4) | | | 1.43 (33) | | | .162 | | | 80.4 (8.5) | | | 75.9 (11.2) | | | 1.17 (33) | | | .250 | | |  |
| Heart rate | | 80.4 (12.1) | | | 77.9 (12.6) | | | 0.53 (25) | | | .602 | | | 85.7 (9.0) | | | 76.9 (12.5) | | | 1.72 (25) | | | .097 | | |  |
| Fasting glucose | | 4.7 (0.4) | | | 4.9 (0.4) | | | -1.16 (22) | | | .258 | | | 4.7 (0.4) | | | 4.8 (0.3) | | | -0.53 (22) | | | .599 | | |  |
| Fasting triglycerides | | 0.96 (0.5) | | | 0.80 (0.23) | | | 0.95 (21) | | | .352 | | | 0.8 (0.2) | | | 0.9 (0.5) | | | -0.67 (21) | | | .513 | | |  |
| Fasting Cholesterol | | 4.2 (0.75) | | | 4.27 (0.67) | | | -0.22 (21) | | | .830 | | | 4.5 (0.6) | | | 4.1 (0.7) | | | 1.25 (21) | | | .225 | | |  |
